# 83. ID Coaches Contribute to a Highly Effective Learning Experience for Third-Year Medical Students Rotating on the Infectious Diseases Consult Service

**DOI:** 10.1093/ofid/ofab466.083

**Published:** 2021-12-04

**Authors:** Erin Roberts, Rachel Sigler, Elliott Welford, Jocelyn Keehner, Darcy Wooten

**Affiliations:** 1 UCSD, San Diego, California; 2 Division of Infectious Diseases, University of California, San Diego, San Diego, CA

## Abstract

**Background:**

Education on infections in hospitalized patients, antimicrobial selection, and principles of antimicrobial stewardship are foundational to all clinicians. Incorporating early learners into Infectious Diseases (ID) consult services has the potential to build a strong fund of knowledge in these content domains, but also poses potential challenges. We evaluated the impact of a novel clinical rotation and supporting curriculum on third-year medical students rotating on the ID consult service for 2 weeks during their 12-week Internal Medicine clerkship at the University of California, San Diego.

**Methods:**

Third-year medical students who selected to rotate on the ID consult service were given an hour-long orientation about the service and common infectious syndromes. They were provided with a checklist of clinical skills to complete during the rotation. In addition to daily rounds and clinical care, ID Coaches (ID faculty and senior ID fellows) met with students weekly for 1-2 hours to review ID topics, practice oral presentations, and/or conduct physical exam finding rounds. We surveyed medical students to assess the effectiveness of the rotation.

**Results:**

Forty third-year medical students participated in the 2-week ID consult rotation between June 2020-May 2021; 31 (77%) completed the rotation evaluation. Seventy percent or more of students reported that the ID rotation facilitated their learning across 8 of 10 ID-content domains (Figure 1). More students reported that the ID Coach facilitated learning (71%) compared to the clinical skills checklist (42%). Students highlighted learning about antimicrobial selection, stewardship, and clinical reasoning on the rotation but reported that teaching was limited when the service census was high (Figure 2).

Figure 1: Percent of Students Rating the ID Consult Rotation as Extremely or Very Effective in Facilitating Learning Across 10 Domains

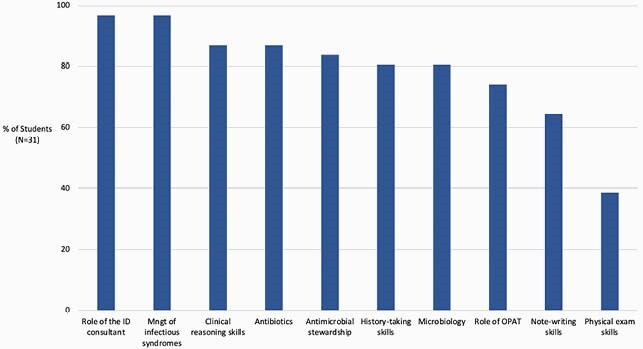

Figure 2: Students' Reflections on the Effectiveness of the ID Consult Rotation

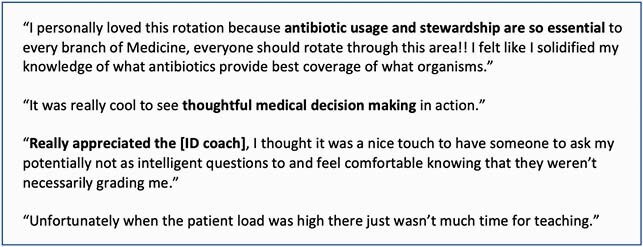

**Conclusion:**

Third-year medical students found that a 2-week rotation on the ID consult service was highly effective in teaching foundational ID content and general medicine skills. Incorporating early learners into a busy and complex subspecialty consult service can be facilitated through the use of supplemental curricular tools such as ID Coaches.

**Disclosures:**

**Darcy Wooten, MD, MS**, Nothing to disclose

